# Dietary folic acid intake, 13 genetic variants and other factors with red blood cell folate concentration in pregnancy-preparing population

**DOI:** 10.1007/s00394-024-03474-z

**Published:** 2024-08-17

**Authors:** Wennan He, Yi Zhang, Xiaotian Chen, Yalan Dou, Yuanchen He, Weili Yan

**Affiliations:** 1 Department of Clinical Epidemiology & Clinical Trial Unit (CTU), Children’s Hospital of Fudan University, National Children’s Medical Center, 399 Wanyuan Road, Shanghai, 201102 P.R. China; 2 Research Unit of Early Intervention of Genetically Related Childhood Cardiovascular Diseases (2018RU002), Chinese Academy of Medical Sciences, Shanghai, P.R. China; 3 Shanghai Key Laboratory of Birth Defect, Children’s Hospital of Fudan University, Shanghai, P.R. China

**Keywords:** Folate, MTHFR, Red blood cell folate, Pregnancy-preparing population

## Abstract

**Purpose:**

This study aims to evaluate a combined effect of dietary folic acid intake, multiple genetic polymorphisms in folate metabolism, and other environmental factors on red blood cell (RBC) folate concentration in pregnancy-preparing population.

**Methods:**

519 pregnancy-preparing subjects (260 couples) were investigated. Dietary intake was measured by 3-day dietary recalls. 13 Single Nucleotide polymorphisms (SNPs) reported in association with one-carbon metabolism including the methylenetetrahydrofolate reductase (MTHFR) C677T and A1298C were genotyped. RBC folate concentration was measured using chemiluminescence assay. Hierarchical regression was applied for covariate selection. Factors showed significance(*p* < 0.0125) on RBC folate level was included for prediction model construction and R^2^ estimation. Validation cohort analysis was performed as post-hoc analysis if applicable.

**Results:**

The median RBC folate was 212.8 ng/ml. Only 10% took folic acid supplementation within three months. Based on hierarchical selection, folic acid supplementation, genetic polymorphism (especially TT genotype of MTHFR C677T), serum folate level were determinants of the variance of RBC folate concentrations, with adjusted R^2^ of 0.178–0.242. MTHFR A1298C polymorphism, sex difference with other socio-demographic and lifestyle factors (age, BMI, alcohol drinking, smoking, education, occupation) explained little to change in RBC folate level. Validation in another sub-cohort(*n* = 8105) had adjusted R^2^ of 0.273.

**Conclusion:**

In pregnancy-preparing subjects, folic acid supplementation, serum folate level and TT allele of MTHFR C677T polymorphism were determinants of the total variance of RBC folate level, which explained 19.8% variance in our subjects and 27.3% in the validation cohort. Food folate intake, sex and other environmental factors explained little to RBC folate level.

**Supplementary Information:**

The online version contains supplementary material available at 10.1007/s00394-024-03474-z.

## Introduction

Folate is a water-soluble B vitamin that is naturally present in foods, or added in fortified foods or dietary supplements, primarily via the form of folic acid [1]. As a core nutrient of one-carbon metabolism, its essential role in fetus development as a coenzyme or co-substrate in single-carbon transfers during the synthesis and methylation of nucleic acids has been well established [1–4]. Achieving sufficient periconceptional folate is known to have profound implications for primary prevention of neural tube defect (NTD)- and congenital heart disease, not only as to maternal folate, but also paternal folate [5–9]. Therefore, investigating folate status and its determinants of pregnancy-preparing population using reliable biomarkers is highly required [1]. However, limited data is available regarding this population.

Red blood cell (RBC) folate reflects the long-term folate status and is the gold-standard measure recommended by the World Health Organization (WHO) [10]. RBC folate concentrations showed high heterogeneity across populations of different food fortification policy and genetic or environmental features [1]. Based on our previous cross-sectional study and the WHO recommendation, > 90% of pregnancy-preparing couples had RBC folate insufficiency (< 906 nmol/L), with 18.9% of females and 32.2% of males having RBC folate deficiency (< 340 nmol/L) [11]. The study summarized socio-demographic variables as potential factors determining folate insufficiency, while no dietary or genetic factors were included, which has been noted as main limitations [11]. A more comprehensive picture describing variation in RBC folate is of particular interest, in settings where periconceptional folic acid supplement use is low and folic acid fortification is not available.

Folic acid intake plays a dominant role in increasing blood folate concentration [3, 12, 13]. Previous dose-response meta-analysis showed a 6% (95% CI: 4%, 9%) increase in RBC folate concentration can occur for every 10% increase in natural food folate intake [14]. Another estimated a median of 36 weeks (95%CI: 27,52) of folic acid intake (375–570 µg /day) to achieve steady-state RBC folate concentrations [15]. Unique contributions of natural food folate and supplemental folic acid on RBC folate should be highlighted to inform the identification of NTD risk among populations with limited awareness of periconceptional folic acid intake. More importantly, gene-nutrient associations should be taken into consideration, as to whether folic acid intake increases RBC folate differed by genotype [16]. Genome-wide association studies (GWAS) have identified several single nucleotide polymorphisms (SNPs) linking to altered blood folate concentration or NTDs through one-carbon metabolism, such as polymorphism in methylenetetrahydrofolate reductase (MTHFR) [16–20]. However, the effect of MTHFR polymorphism on blood folate concentration showed varied patterns differed by biomarkers and methodology [16–18]. Evidence was scarce for other SNPs, and no combined effect of genetic variants was investigated in terms of RBC folate concentration. Conflicting findings were also found in other socio-demographic and lifestyle factors, such as age, sex and smoking [11, 21, 22]. Comparison between studies were still lacking and to what extent these explain variation in RBC folate remains to be elucidated.

To better understand this issue, we used data from a sub-cohort of pregnancy-preparing couples with several dietary and environmental factors, and a total of 13 SNPs. This study aims to identify critical factors that explained variation in RBC folate, as well as evaluate a combined effect of dietary folic acid intake, multiple genetic polymorphisms in folate metabolism, and other environmental factors on red blood cell (RBC) folate concentration in pregnancy-preparing population.

## Methods

### Participants

This study analyzed data from a sub-cohort nested in the ongoing birth cohort, the Shanghai Preconception Cohort (SPCC). Briefly, SPCC was initially established to investigate the effect of parental periconceptional nutritional factors on congenital heart disease since 2016, with extension to other birth defects, child growth and development [23]. Couples attending pre-pregnancy physical examinations at Minhang district in Shanghai between March 2018 and November 2018, and met the following criteria were eligible for this cross-sectional study: (1) aged 18–45 years at enrollment; (2) planning for pregnancy within 6 months; (3) free of any genetic disorders, psychiatric diseases, hemorrhagic or hemolytic diseases, cardiovascular diseases, liver or kidney diseases, and did not have any infectious diseases recently; (4) did not take antineoplastic or antipsychotic drugs within 3 months; (5) able to follow instructions and complete the questionnaire.

Eligible participants were invited to complete 3-day dietary recalls at enrollment in this sub-cohort. Questionnaires and blood samples were collected following the whole SPCC data collection procedure. This study has been approved by the Ethics Committee of the Children’s Hospital of Fudan University, Shanghai, China (Institutional Review Board No.2016-49). Written informed consent was obtained from all participants before enrollment.

### Data collection

Dietary assessment was performed by an independent trained dietitian using consecutive 3-day 24-hour dietary recall. Portion-size food molds were used to assess the serving sizes. To calculate dietary intakes of folate and the relevant nutrients, the nutrient composition of each food was multiplied by the total quantity of food consumption and then summed over all food items based on Chinese Food Composition Table [24].

Data on socio-demographic characteristics, lifestyle factors were obtained through self-reported questionnaires. Trained coordinated staffs provided necessary instructions during face-to-face interviews and filled in the well-constructed questionnaires. Folic acid supplementation was recorded in detail, including the supplement brand name, daily dose, and how often the participants used supplements every month.

Venous blood samples were collected at enrollment using light-proof tubes and treated within 6 h by trained nurses following SPCC standard procedure. Samples were stored temporarily in a − 20◦C refrigerator at recruiting sites, shipped on dry ice weekly during transferring and stored in -80◦C refrigerator in the central laboratory of the Children’s Hospital of Fudan University for further biomarker assessment. Details of blood collection and storage could be found elsewhere [23]. Dietary assessment, questionnaires and blood collection of the same participant were all completed on the same day at enrollment.

### Biomarker assessment

Details of quality control on biomarker assessment were described in the previous SPCC papers [11, 23]. RBC folate, serum folate (in the form of 5-methyltetrahydrofolate), serum homocysteine, vitamin D, vitamin B12 and serum ferritin were measured in the central laboratory of the Children’s Hospital of Fudan University. All six biomarkers were analysed using electro-chemiluminescent competitive protein binding assays (ARCHITECT i2000SR Analyzer; Abbott Laboratories, USA). External quality control was conducted with the control laboratory data programme from Abbott Laboratories (Abbott Laboratories, Shanghai, China).

### Genomic DNA extraction and genotyping

Genomic DNA was extracted from 2 mL of EDTA anticoagulated whole blood sample using a magnetic bead-­based kit (TGuide M16 Automatic Nucleic Acid Extractor (OSE-­M16); Tiangen Biotech (Beijing) Co., China).

The genotyping of 8 key single nucleotide polymorphisms (SNPs) reported in association with one-carbon metabolism, and/or circulating folate and homocysteine, for MTHFR 677 C/T (rs1801133) and 1298 A/C (rs1801131), MTR 186 T/G(rs28372871) and 2756 A/G (rs1805087), MTRR 66 A/G (rs1801394) and c.56 + 781 A/C(rs326119), MTHFD1 1958 G/A(rs2236225), BHMT G/A(rs3733890) polymorphisms were performed using the TaqMan allelic discrimination assay on the platform of QuantStudio Real-Time PCR software (Applied Biosystems) with standard quality control. Between November 2022 and January 2023, genotyping of 5 other SNPs for FIGN C/G (rs2119289), SHMT G/A (rs1979277), RFC1 T/C (rs1051266), MTHFR G/A (rs3737965), MTR G/A (rs1131450) was further performed using the remaining blood samples.

Allelic and genotype frequencies for each SNP were based on reference value in East-Asian population in 1000Genomes study using online database [25]. SNPs genotyping failed in some participants because of a limited sample volume. Detailed SNPs information can be found in Table S1.

### Potential covariates

Data on Socio-demographic factors (age, sex, BMI, education level, occupation, smoking and alcohol drinking status), folate intake (food folate intake, total energy intake and folic acid supplementation), serum folate(ng/ml) and SNPs were chosen priori as candidate covariates affecting RBC folate level. Age was measured in years. BMI was calculated as weight in kilograms divided by height in meters squared (kg/m^2^). Education levels were categorized as high school or lower, diploma or bachelor and postgraduate degrees. Occupations were categorized as blue collar, white collar, farmer and others. The status of smoking, alcohol drinking and folic acid supplementation was summarized as binary (yes or no) within three months before enrollment. Total energy intake was included for dietary folate adjustment using the residual method [26]. Daily intake of total energy (kcal/d) and dietary folate(mg/d) was obtained by averaging the total amount from 3-day dietary recall. Food folate intake was also summarized as different threshold levels by every 100 mg/d increase. Dietary folate equivalent was obtained by multiplying the average supplemental folic acid intake by 1.7 and added to dietary folate intake. SNPs were coded as per risk allele increased (i.e. 0 for homozygote with the common allele, 1 for the heterozygote, and 2 for homozygote with the rare allele). The weighted joint effect of 8 SNPs and 13 SNPs were calculated by using Polygenic genetic risk scores (PG-GRS) [27], as the sum of weights for each allele corresponding to estimated genetic effect sizes of the associations between the SNP and RBC folate [28]. Because MTHFR 677 C/T (rs1801133) and 1298 A/C (rs1801131) are two genetic variants that showed most consistency in terms of the effect on blood levels of folate [16–19], these two were chosen as an independent covariate other than PG-GRS.

### Statistical analysis

Descriptive statistics were performed in total and by sex. Characteristics of study participants was expressed as the mean (standard deviation) for normally distributed variables, median (inter-quartile range) for skewed variables and frequency with percentage for categorical variables. Group comparisons were performed by two-sample t-test, Kruskal Wallis test and chi-square test respectively. P-value of < 0.05 was considered as significant group difference. Validation of blood biomarkers was assessed by pairwise correlations (Table S2).

Hierarchical linear regression was applied for covariate selection as RBC folate concentration being the dependent variable. Before fitting the model, assumptions that underpin multiple regression and linearity were tested as priori. Serum and RBC folate concentration was log-transformed to reach the normal distribution. RBC folate concentration was further normalized as the z-score within the sub-cohort for generalizability in the whole cohort. Dietary folate was included as categorical variable (1,>=100and < 200; 2, >=200and < 300; 3,>=300and < 400; 4,>=400). Unfortunately, dietary folate equivalent was not included for analysis because we found the supplemental folic acid intake was highly inaccurate as most of them did not take the supplementation or take it occasionally that had a poor memory about the true frequency of consumption. Thus, folic acid supplementation use (yes or no) was included instead.

**Fig. 1:**
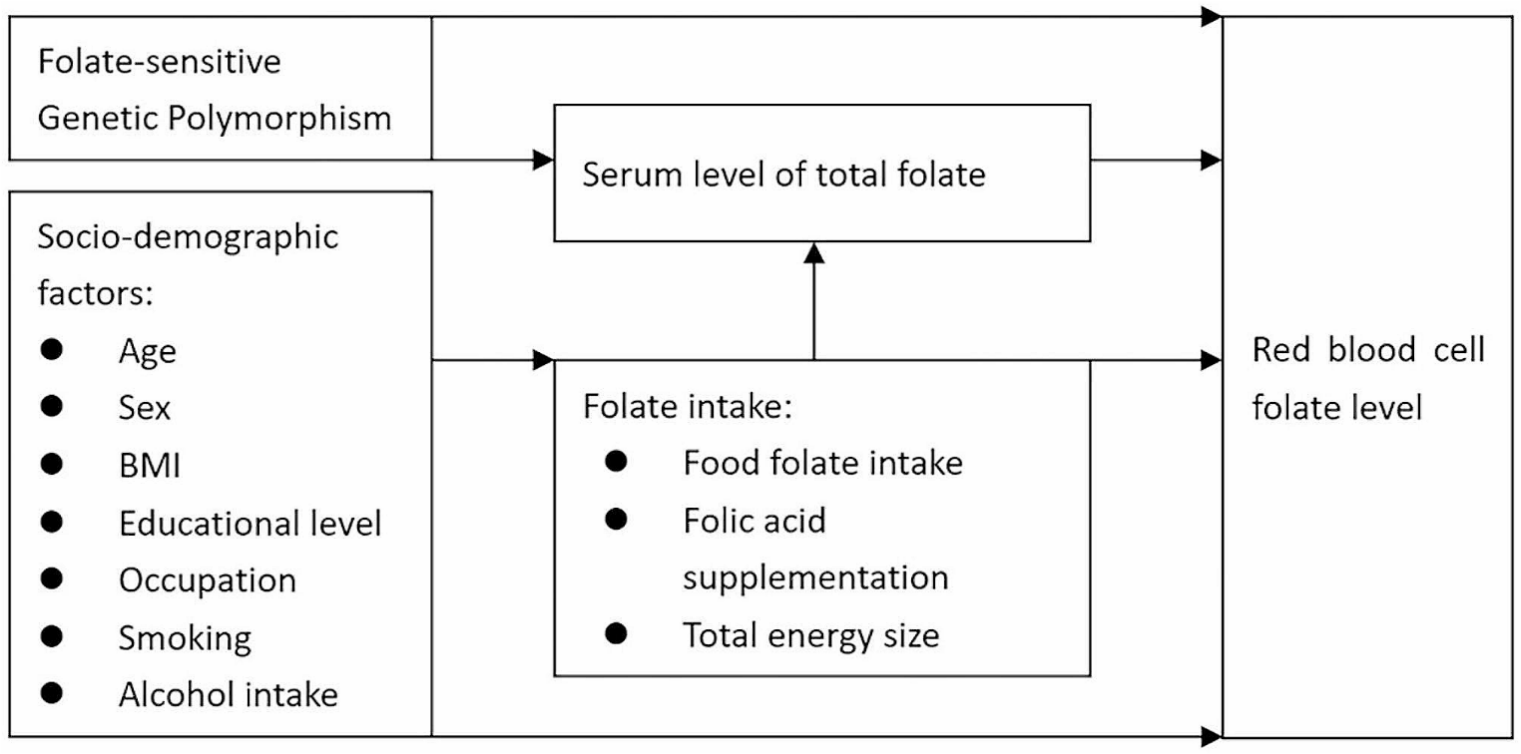
Theoretical framework of factors associated with RBC folate level in this study

Hierarchical regression analysis allowed hierarchical selection for determining the forward inclusion of covariates [29]. Before then, a theoretical framework, including four blocks of factors associated with RBC folate level (Fig. 1) has been built based on the authors’ background knowledge and previous similar frameworks [30, 31]. The contribution of four blocks of covariates was identified step by step in explaining RBC folate level. The significant level was set at 0.05/4 = 0.0125, because four hierarchical regression models were performed during forward inclusion of blocks (block 1; block 1 + 2; block 1 + 2 + 3; block 1 + 2 + 3 + 4). Socio-demographic factors were entered as covariates in the first block, factors of folate intake were placed in the second block. Serum level of folate was included in the model as the third block and folate-sensitive genetic polymorphisms were the fourth block. Within the block, the variables were entered simultaneously. By fitting additional regression models, R-squared were used for comparisons between models to see if the latter performed better than the former model. After covariate selection, generalized linear model was used for prediction, as hierarchical regression only allowed continuous variables that might mask the true contribution from specific category in factor variables. A post-hoc analysis including generalized linear model and Bland-Altman plot [32, 33] was used for validation in another sub-cohort, which recruited participants from Kunshan city in China, if applicable.

To further check consistency of the findings, we considered a sex-stratified analysis, as well as missing data handling. We hypothesized a missing at random(MAR)missingness mechanism for incomplete data. Multiple imputation(MI) using chained equations (arbitrary missing pattern with 10 imputations) on missing data was conducted as sensitivity analysis for the main generalized linear models after covariate selection. The MI analyses were conducted applied to all the chosen covariates in the analysis model, imputed using either linear or ordered logistic regression, as applicable, dependent on other remaining covariates excluded from hierarchical selection.

We did not adjust for multiple testing in this study. The data analysis was performed through STATA (Version 15.1, StataCorp L.P., and College Station, TX).

## Results

### Characteristics of the participants

Table 1 shows the general characteristics of study population. 519 subjects were eligible for analysis, with 259 males and 260 females. Overall, the characteristics was similar with another large SPCC pre-pregnancy cohort [11]. Participants were 29 years and had BMI of 22.8 in average. The mean dietary folate intake was 296.9 mg per day. The median RBC folate was 212.8 ng/ml and serum folate was 5.7 pg/ml. Over 90% participants received higher education and nearly 85% were white collar. 118(22.7%) and 184(35.5%) reported history of smoking and alcohol drinking within three months respectively.

**Table 1:** General characteristics of the study population

	Total (n = 519)	Male(n = 259)	Female(n = 260)	P
**Age(years)** ^a^	29(4)	30(4)	28(3)	< 0.001
**BMI (kg/m**^2^)^a^	22.8(3.1)	24.6(2.9)	21.0(2.2)	< 0.001
**Food folate intake(mg/d)** ^a^	296.9(66.7)	327.3(63.2)	266.7(55.4)	< 0.001
**Total energy intake(kcal/d)** ^b^	2574(447)	2805(358)	2345(407)	< 0.001
**RBC folate(ng/ml)** ^b^	212.8(151.8,310.7)	194.9(149.0,284.1)	229.5(164.8,334.2)	0.0015
**Serum folate(ng/ml)** ^b^	5.7 (4.0,8.5)	4.8(3.5,6.7)	6.9(4.8,9.7)	< 0.001
**Vitamin B12 (pg/ml)** ^b^	402(305,531)	369(291,439)	461(333,595)	< 0.001
**Homocysteine (umol/L)** ^b^	6.6(5.1,8.5)	7.8(6.5,9.6)	5.5(4.5,6.9)	< 0.001
**Educational level, n(%)**				< 0.001
High school or lower	46(8.9)	9(3.4)	37(14.2)	
Diploma or bachelor	280(53.9)	125(48.3)	155(59.6)	
Postgraduate	193(37.2)	125(48.3)	68(26.2)	
**Occupation, n(%)**				0.289
Blue collar	30(5.8)	18(7.0)	12(4.6)	
White collar	442(85.2)	221(85.3)	221(85.0)	
Farmer	2(0.4)	0(0)	2(0.8)	
Others	45(8.6)	20(7.7)	25(9.6)	
**Smoking(yes), n(%)**	118(22.7)	88(34.0)	30(11.5)	< 0.001
**Alcohol drinking(yes), n(%)**	184(35.5)	120(46.3)	64(24.6)	< 0.001
**FA supplementation** **within three months (yes), n(%)** ^c^	60(11.6)	10(3.9)	50(19.2)	< 0.001
**MTHFR A1298C, n(%)**				< 0.001
AA	351(67.6)	180(69.5)	171(65.8)	
CA	111(21.4)	37(14.3)	74(28.5)	
CC	40(7.7)	32(12.3)	8(3.1)	
Undetermined	17(3.3)	10(3.9)	7(2.7)	
**MTHFR C677T, n(%)**				0.579
CC	162(31.2)	79(30.5)	83(31.9)	
CT	247(47.6)	123(47.5)	124(47.7)	
TT	92(17.7)	40(15.4)	52(20.0)	
Undetermined	18(3.5)	17(6.6)	1(0.4)	
**GRS of 8 SNPs** ^a, d^	35.5(89.7)	29.5(85.3)	40.9(93.3)	0.197
**GRS of 13 SNPs** ^a, e^	65.6(111.6)	57.6(100.3)	73.2(121.3)	0.252

*Abbreviation* BMI, body mass index; RBC, Red Blood Cell; FA, Folic Acid; MTHFR, methylenetetrahydrofolate reductase; GRS, genetic risk score

^a^ summarized as mean (standard deviation)

^b^ summarized as median (p25, p75)

^c^ Among those taking supplementations, 58 took 400 µg folic acid/d and 2 took 500–800 µg folic acid/d

^d^ SNPs include MTHFR 677 C/T (rs1801133) and 1298 A/C (rs1801131), MTR 186T/G(rs28372871) and 2756 A/G (rs1805087), MTRR 66 A/G (rs1801394) and c.56 + 781 A/C(rs326119), MTHFD1 1958G/A, BHMT G/A(rs2236225)

^e^ SNPs further include FIGN C/G (rs2119289),SHMT G/A (rs1979277), RFC1 T/C (rs1051266), MTHFR G/A (rs3737965), MTR G/A (rs1131450)

There was a significant sex difference almost in all characteristics, especially in folate intake, folic acid supplementation, biomarker concentrations, smoking and alcohol drinking status. Males had a higher energy intake with higher food folate intake than females (2805 ± 358 kcal vs. 2345 ± 407 kcal per day; and 327.3 ± 63.2 mg vs. 266.7 ± 55.4 mg per day). A detailed dietary nutrient profile was summarized and almost all nutrient intakes were higher in males than females (Table S6). 60(11.6%) subjects reported folic acid supplementation within three months, with 50 of them were females. Females had higher levels of RBC folate, serum folate, vitamin B12, and lower level of homocysteine than males. Most of smokers and alcohol drinkers were males, as 88(34.0%) males reported smoking and 120(46.3%) reported alcohol drinking within three months.

### Hierarchical selection of potential covariates

The results of hierarchical selection of potential predictive factors influencing RBC folate level are shown in Table 2, which briefly demonstrates the corresponding indexes during first forward inclusion of variables within each block. Socio-demographic factors contributed 2.8% of variance (i.e. R^2^ of 0.028), and sex was the significant predictor of RBC folate level in block 1(*P* = 0.002). However, sex became non-significant after including other blocks (all *p* > 0.1, data not shown). In block 2, food folate intake showed no significant effect on RBC folate level(*p* = 0.948), while folic acid supplementation was a significant indicator of RBC folate level (*p* < 0.001). Serum folate was significant when included as block 3(*p* < 0.001), and explained variance increased to 13.2% compared to 5.8% of block 1 + 2. MTHFR C677T(*P* < 0.001), but not MTHFR A1298C(*p* = 0.147), showed significance influencing RBC folate level. The total explained variance increased to 17.8%. GRS of 8 SNPs and 13 SNPs showed significance(*p* < 0.001) and slightly contributed greater explained variance of RBC folate level, compared to MTHFR C677C alone, with 24.2% in 8 SNPs and 20.4% in 13 SNPs.

**Table 2:** Hierarchical selection of potential predictive factors influencing RBC folate level

	Beta	Std. Err.	t	P>|t|	R-squared	Change in R-squared
**Block 1: Socio-demographic factors**					0.028	
Age	0.017	0.011	1.59	0.112		
Sex	0.351	0.111	3.15	0.002		
BMI	0.004	0.017	0.24	0.814		
Education	0.080	0.074	1.07	0.283		
Occupation	-0.015	0.022	-0.67	0.501		
Smoking	0.037	0.109	0.34	0.732		
Alcohol	0.041	0.095	0.43	0.668		
**Block 2: Folate intake**					0.058	0.030
Food folate ^a^	-0.005	0.072	-0.06	0.948		
FA supplementation	0.554	0.139	3.98	< 0.001		
Total energy intake	-0.00008	0.0001	-0.69	0.492		
**Block 3: Serum level of total folate**					0.132	0.074
Serum folate^b^	0.576	0.088	6.51	< 0.001		
**Block 4: Genetic Polymorphism** ^c^						
MTHFR C677T (*n* = 495)	0.352	0.061	5.78	< 0.001	0.178	0.047
MTHFR A1298C (*n* = 496)	-0.103	0.068	-1.45	0.147	0.134	0.002
GRS of 8 SNPs (*n* = 408)	0.003	0.0005	6.62	< 0.001	0.242	0.111
GRS of 13 SNPs (*n* = 267)	0.003	0.0005	4.84	< 0.001	0.204	0.072

Dependent variable: Red blood cell folate level (log-transformed and standardized)

*Abbreviation* BMI, body mass index; FA, Folic Acid; MTHFR, methylenetetrahydrofolate reductase; GRS, genetic risk score

^a^Food folate was transferred into categorical variable, coded as 1, >=100 and  <200;2, >=200 and  <300;3, >=300 and  <400;4, >=400

^b^Serum folate was log-transformed

^c^Four different sets of genetic polymorphism were included in the hierarchical regression model separately due to varied sample size; GRS of 8 SNPs: genetic risk score of 8 SNPs (rs1801131, rs1801133, rs28372871, rs1805087, rs1801394, rs326119, rs2236225, rs3733890); GRS of 13 SNPs: genetic risk score of 13 SNPs (rs1801131, rs1801133, rs28372871, rs1805087, rs1801394, rs326119, rs2236225, rs3733890,rs2119289, rs1979277,rs1051266, rs3737965, rs1131450)

### Predictive factors influencing RBC folate level

Covariates showed consistent significance explaining RBC folate level (folic acid supplementation, serum folate and genetic polymorphisms) were included simultaneously in the generalized linear model (Table 3). Folic acid supplementation (0.270,95%CI:0.009,0.533) and serum folate (0.660,95%CI:0.493,0.826) showed effects on RBC folate level in the sub-cohort. Subjects with TT allele of MTHFR C677T contributed to RBC folate level compared to those with CC allele (0.787, 95%CI:0.553,1.021). No significant difference was observed in terms of CT allele.

**Table 3:** Predictive factors influencing RBC folate level

	Beta(95%CI)	t	P>|t|
**Sub-cohort(n = 495):**			
**FA supplementation**			
1.Yes (vs. 0.No)	0.270(0.009,0.533)	2.03	0.043
**Serum folate**(_log_ug/ml)	0.660(0.493,0.826)	7.80	< 0.001
**MTHFR C677T**			
1.CT (vs. 0.CC)	0.014(-0.167,0.194)	0.15	0.882
2.TT (vs. 0.CC)	0.787(0.553,1.021)	6.61	< 0.001
**Cons.**	-1.353(-1.692,-1.014)	-7.85	< 0.001
**R-squared**	0.204		
**Adjusted R-squared**	0.198		
**Validation cohort (n = 8105):**			
**FA supplementation**			
1.Yes (vs. 0.No)	-0.303(-0.487,-0.118)	-3.22	0.001
**Serum folate**(_log_ug/ml)	0.980(0.941,1.020)	48.83	< 0.001
**MTHFR C677T**			
1.CT (vs. 0.CC)	-0.043(-0.086,0.0005)	-1.94	0.052
2.TT (vs. 0.CC)	0.758(0.706,0.811)	28.18	< 0.001
**Cons.**	-2.030(-2.116,-1.944)	-46.09	<0.001
**R-squared**	0.273		
**Adjusted R-squared**	0.273		

Dependent variable: Red blood cell folate level (log-transformed and standardized)

*Abbreviation* FA, Folic Acid; MTHFR, methylenetetrahydrofolate reductase

The included variables explained an unadjusted 20.4% and adjusted 19.8% of total variance in RBC folate level in the sub-cohort. Using GRS yielded similar results as to MTHFR C677T, as explained an adjusted 22.1% of total variance for 8 SNPs, and 18.1% for 13 SNPs (Table S3). Sex-stratified analysis showed similar results as above. Multiple imputation on missing data (RBC folate, *n* = 2;serum folate, *n* = 6;MTHFR C677T, *n* = 18) also provided consistent findings (Table S4).

### Validation cohort analysis

Data of the validation cohort we used was updated on May 2024, with complete data on folic acid supplementation (yes or no), serum and RBC folate level, MTHFR C677T polymorphism. Results in the validation cohort(*n* = 8105) were similar with that in the sub-cohort (Table 3). The effect size of folic acid supplementation became more significant in the validation cohort (-0.303, 95%CI: -0.487, -0.118). Serum folate (0.980,95%CI: 0.941,1.020) and subjects with TT allele of MTHFR C677T compared to CC allele (0.758,95%CI:0.706,0.811) showed positive associations with RBC folate. CT allele showed minimally negative effect in the whole cohort (-0.043,95%CI: -0.086,0.0005). The total variance on RBC folate increased to 27.3% explained by above variables in the validation cohort.

**Fig. 2:**
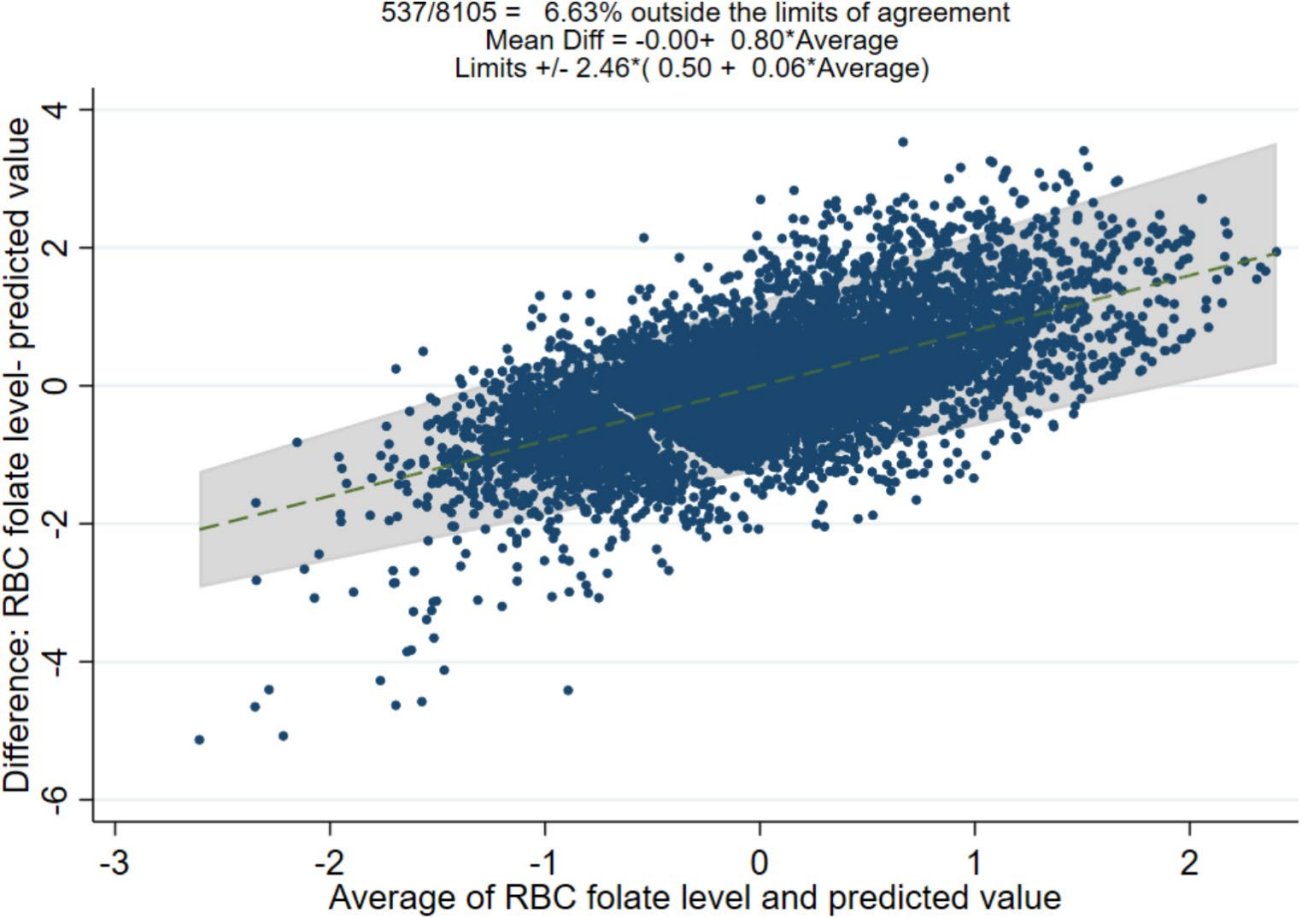
Bland-altman plot assessing the agreement between actual values and predicted values of RBC folate level in the validation cohort (N = 8105). The plot is constructed using horizontal V-shaped 95% confidence limits because of proportional bias and heteroscedasticity [1]. (- - -) dashed line: ordinary least squares (OLS) line of best fit; grey scale: area covering upper and lower 95% confidence limits. On OLS regression, Mean difference = -0.00 + 0.80* Average. Reference: [1]. Ludbrook, J., Confidence in Altman-Bland plots: a critical review of the method of differences. Clin Exp Pharmacol Physiol, 2010. 37(2): p. 143-9

Figure 2 shows a Bland-Altman plot assessing the limits of agreements between actual values and predicted value of RBC folate level. The predicted value was derived from the above generalized linear model in the validation cohort. Bland-Altman prediction estimated 537 out of 8105 subjects (6.63%) were outside the limits of agreement. On ordinary least squares (OLS) regression, Mean difference = -0.00 + 0.80* (Average), with limits of ± 2.46*(0.50 + 0.06*Average).

## Discussion

To our knowledge, this is the first study targeting pregnancy-preparing couples to assess a combined effect of 13 SNPs, dietary and other environmental factors with circulating serum folate on variation in long-term folate status. We found that compared to natural food folate intake and other socio-demographic factors, folic acid supplementation, serum folate level and carrying TT allele of MTHFR C677T showed independent effects on RBC folate level, with total variance of 17.8% in the sub-cohort and further validated as 27.3% in the validation cohort. Using GRS of 8 SNPs slightly increased the variance to 24.2% while 13 SNPs did not. The hierarchical selection of critical factors that uniquely contributing to RBC folate level might provide a better understanding on the role of these factors on a long-term folate status in pregnancy-preparing population.

Since 2015, WHO recommends a daily intake of 400 µg folic acid for women of reproductive age to prevent NTDs [10]. Higher level of folic acid intake might further reduce risks of congenital diseases [7, 34]; however, increasing folate level through natural food folate alone is challenging and dietary habit might be kept as usual even in pregnancy-preparing couples. This could be reflected by averagely low, and energy-dependent food folate intake in our subjects. The importance of folic acid supplementation is accordingly highlighted in population without folic acid fortification. We found a positive effect of taking folic acid supplementation on RBC folate, showing consistency with previous studies [11, 35]. The effect size was attenuated in generalized linear model, possibly due to its high influence on altering serum folate level. Given that serum folate level is sensitive to recent dietary intake [1], the effect of dietary factors might not effectively reflect the long-term folate status. Discrepancy between self-reported diet and folate biomarkers was also influenced by different populations, and subject to measurement issues [36]. Nevertheless, we observed folic acid supplementation significantly increased RBC folate level, regardless of duration or frequency of consumption. Unmeasured physiologic or pathologic exposures to synthetic folic acid might help explain elevated folate concentration in RBC and the complexity with other circulating biomarkers deserves further exploration. In brief, similar to previous suggestions [15, 35, 37], compared to food source, our findings indicated any folic acid supplements might be beneficial for improvement on long-term folate status.

Polymorphism’s impact on NTD risk is greater in populations with lower folate intakes, through change in RBC folate concentration, showing most consistency on MTHFR C677T [17, 38]. This was further confirmed in our study, that genetic polymorphism played an independent role in altering RBC folate level. Using PG-GRS of 8 SNPs and 13 SNPs contributed to nearly half of variance among all candidate covariates. However, a weighted combination of multiple variants did not show superiority over MTHFR C677T with a CC, CT, TT pattern. MTHFR C677T did not follow a dose-response trend as per risk allele increase, but showed significance only in subjects carrying TT allele. This might be explained by massive cellular non-methylfolate accumulation in subjects with TT genotype compared to CC and CT genotype, as reported before [39, 40]. The different propensity to non-methylfolate accumulation seemed more obvious in low-dose folate status and unaffected by serum folate level [40]. Otherwise, it might be masked by differential methods. Compared with microbiologic assay (MA), protein-binding assays (PBAs) underestimates whole-blood folate concentrations in individuals with CC and CT genotypes by an average of 43–46%, but only by 26–35% in individuals with the TT genotype (who have greater proportions of non–5-methyl-THF species) [41]. This led to a reversed CC, CT, TT whole-blood folate pattern, as a higher RBC folate level was detected followed by per 1 risk allele increased [16]. The reversed pattern between TT genotype with RBC folate was constant in healthy population, no matter low or high folic acid intake [42]. Although it affected little to the total variance explained by genotyping, our results should be interpreted with caution and similar reversed patterns with other SNPs might occur.

Dozens of studies have summarized the effect of parental folate on offspring health [43], while few focused on sex difference on altering folate status and corresponding health risks. Due to sex steroids and growth hormone secretion, sex-differential enzyme expressions in one-carbon metabolism are reported to affect metabolites levels and modified by genetic polymorphism [44, 45]. Theoretically, females have lower S-andenosylmethionine, lower homocysteine, and higher choline and betaine, accordingly follow a different pattern of folate species compared to males [44]. However, we did not observe significant sex difference on altering RBC folate level. The total variance explained by socio-demographic factors was only 2.3%, and sex became no longer significant afterwards. The sex-stratified analysis also showed similar findings. Previous cross-sectional studies showed mixed results and left a question regarding how sex involved in obscure associations between lifestyle factors with folate status [46–48]. One of the possibilities, as we mentioned before [11], is the differential socio-demographic features and lifestyle habits between Chinese females and males, which are further enhanced during pregnancy-preparing. Based on our models, most of these differences might have been reflected by change in dietary supplementation use and circulating biomarkers, which eventually contribute to long-term folate status. We are not arguing sex with other socio-demographic or lifestyle factors have no effect on altering RBC folate level, but in this setting, moderate characteristics with narrow ranges might have far less significance to long-term folate status, compared to populations with more skewness on certain characteristics (e.g. obesity, age, folic acid fortification, smoking, etc.) [21, 22, 49–52]. Controversies also rise across different scaling of variables that should be interpreted cautiously. Taken together with our previous sex-stratified analysis [11], some lifestyle factors (BMI, alcohol drinking, smoking) might have effect on RBC folate level; however, the effect seemed not as large as we expected, compared to folic acid supplementation and genetic polymorphism.

Followed by our previous investigation in pregnancy-preparing couples [11], this study included a wider coverage of genetic and dietary factors. We were able to further identify what, and to what extent these determinants alter RBC folate level in this population with a real-world setting. We got a clearer picture of unique contributions among multiple factors during step-by-step hierarchical selection, and supported by the validation cohort. However, as we noticed, this study might narrow the gap in terms of relative importance on folate status in pregnancy-preparing subjects, the results need cautious interpretation due to some obvious limitations behind. First, some SNPs failed in genotyping due to lack of sample volume, which might affect model indexes. Limited sample size could lead to underestimation of actual effect in some SNPs. Despite this, the distribution of undetermined genotypes was more likely to be random and genotype frequencies were similar to other Chinese population [53]. Second, some measurement issues, such as PBAs might obscure the true associations on RBC folate concentration. Self-report diets and unmeasured physiologic and pathologic factors also increased inaccuracy of the results. Third, we standardized RBC folate to increase generalizability across cohorts, while it made the interpretation more challenging. Similar issue comes to the use of Bland-Altman plot. Although a low proportion of disagreement was achieved, the 95%CI was not narrow enough for a standardized value [33]. The plot also increased the possibility of less fit in subjects with extremely high or low RBC folate levels. Fourth, our results might not be generalizable to populations with different folate status and socio-demographic features. Fifth, although the energy-residual method helps deal with other energy-dependent dietary factors not included in the models, we cannot fully exclude the residual confounding or interaction with some certain nutrients on affecting RBC folate. Finally, it was pitiful that dietary data was available in a sub-cohort at a single time point. Also, using 3-day 24-hour dietary recalls might not accurately capture long-term nutrient intakes. In the future, a broader coverage with long-term dietary assessment tool, such as well-quantified domestic FFQ tool and repeated measures might be helpful for us to detect the role of time-dependent factors on long-term folate status in this population. Further investigation is clearly needed to address many unresolved issues such as: (1) in the real-world setting, to what extent modifiable lifestyle factors will have considerable impact on altering long-term folate status, and how they interact with compensating in folate homeostasis; (2) whether and to what extent the risk prediction models on altering RBC folate level differed by MTHFR C677T polymorphism.

In conclusion, folic acid supplementation, serum folate level and genetic polymorphism, especially carrying TT allele of MTHFR C677T polymorphism were determinants to the total variance of RBC folate level in pregnancy-preparing population. The total variance was 19.8% in the sub-cohort and validated as 27.3% in the validation cohort. Using GRS of a total of 13 SNPs related to one-carbon metabolism did not greatly alter the variance compared to MTHFR C677T alone. MTHFR A1298C polymorphism, sex difference with other socio-demographic and lifestyle factors explained little to change in RBC folate level.

## Electronic supplementary material

Below is the link to the electronic supplementary material.

Supplementary Material 1

## Data Availability

Data described in the article, code book, and analytic code will be made available from the corresponding authors upon reasonable application.
