# Supplementary material for: Dietary folic acid intake, 13 genetic variants and other factors with red blood cell folate concentration in pregnancy-preparing population
Source: Eur J Nutr. 2024 Aug 17;63(8):2921–31. doi: 10.1007/s00394-024-03474-z (PMC11519176; doi:10.1007/s00394-024-03474-z)
Supplement: Supplementary file 1 — Supplementary Material 1 [file 394_2024_3474_MOESM1_ESM.docx]

**Supplementary information**

**TableS1. 13 SNPs genotyping Information^1^**

| **SNPs^3^** | **Gene** | **Chromosome Position** | **Nucleotide Change** | **Genotype frequency**  **11/12/22**  **(numbers)^4^** | **MAF^2^** |
| --- | --- | --- | --- | --- | --- |
| rs1801131 | MTHFR | chr1:11794419 | A→C | 351/111/40 | 0.219 |
| rs1801133 | MTHFR | chr1:11796321 | C→T | 162/247/92 | 0.704 |
| rs28372871 | MTR | chr1:236795232 | G→T | 110/259/115 | 0.469 |
| rs1805087 | MTR | chr1:236885200 | A→G | 380/79/3 | 0.105 |
| rs1801394 | MTRR | chr5:7870860 | A→G | 272/177/30 | 0.262 |
| rs326119 | MTRR | chr5:7869970 | A→C | 161/237/74 | 0.376 |
| rs2236225 | MTHFD1 | chr14:64442127 | G→A | 223/182/81 | 0.198 |
| rs3733890 | BHMT | chr5:79126136 | G→A | 236/204/46 | 0.315 |
| rs2119289 | FIGN | chr2:163641436 | C→G | 146/246/88 | 0.213 |
| rs1979277 | SHMT | chr17:18328782 | G→A | 362/40/4 | 0.060 |
| rs1051266 | RFC1 | chr21:45537880 | T→C | 156/213/37 | 0.474 |
| rs3737965 | MTHFR | chr1:11806394 | G→A | 388/78/2 | 0.122 |
| rs1131450 | MTR | chr1:236898549 | G→A | 295/11/13 | 0.221 |

**^1^**derived from online database: <https://www.ncbi.nlm.nih.gov/snp/>

^2^Minor allele frequency (MAF) was referenced based on East-Asian population in 1000Genomes study.

^3^The former 8 SNPs were calculated for genetic risk score of 8 SNPs (i.e. rs1801131, rs1801133, rs28372871, rs1805087, rs1801394, rs326119, rs2236225, rs3733890).

^4^ Numbers of subjects with: 11 for homozygote with the common allele, 12 for heterozygote, and 22 for homozygote with the rare allele.

**Table S2. Pairwise Spearman correlation between dietary folate, energy intake, levels of folate, homocysteine and Vitamin B12**

|  | RBC folate | Serum folate | Vitamin B12 | Homocysteine | Dietary folate | Energy intake |
| --- | --- | --- | --- | --- | --- | --- |
| RBC folate | 1 |  |  |  |  |  |
| Serum folate | 0.3441* | 1 |  |  |  |  |
| Vitamin B12 | 0.0654 | 0.2721* | 1 |  |  |  |
| Homocysteine | -0.1617* | -0.4310* | -0.2052* | 1 |  |  |
| Dietary folate | -0.0008 | -0.1058* | -0.2041* | 0.1327* | 1 |  |
| Energy intake | -0.0598 | -0.1243* | -0.1146* | 0.2817* | 0.3461* | 1 |

* The significance level of <0.05 for correlation coefficients.

**Table S3. Explained variation of RBC folate level using GRS of 8 and 13 SNPs**

|  | Beta(95%CI) | t | P>\|t\| |
| --- | --- | --- | --- |
| GRS of 8 SNPs (n=408): |  |  |  |
| FA supplementation |  |  |  |
| 1.Yes (vs. 0.No) | 0.289(0.001,0.576) | 1.98 | 0.049 |
|  |  |  |  |
| Serum folate(_log_ug/ml) | 0.703(0.497,0.866) | 7.53 | <0.001 |
|  |  |  |  |
| GRS | 0.003(0.002,0.004) | 6.66 | <0.001 |
| Cons. | -1.396(-1.729,-1.06) | -8.23 | <0.001 |
| R-squared | 0.227 |  |  |
| Adjusted R-squared | 0.221 |  |  |
|  |  |  |  |
| GRS of 13 SNPs (n=267): |  |  |  |
| FA supplementation |  |  |  |
| 1.Yes (vs. 0.No) | 0.404(-0.028,0.837) | 1.84 | 0.067 |
|  |  |  |  |
| Serum folate(_log_ug/ml) | 0.638(0.394,0.882) | 5.15 | <0.001 |
|  |  |  |  |
| GRS | 0.002(0.001,0.003) | 5.00 | <0.001 |
| Cons. | -1.356(-1.798,-0.914) | -6.00 | <0.001 |
| R-squared | 0.191 |  |  |
| Adjusted R-squared | 0.181 |  |  |

^1^excluding CBS gene variants (rs2851391 and rs2850144) from GRS.

Dependent variable: Red blood cell folate level (log-transformed and standardized)

*Abbreviation: GRS, genetic risk score; FA, Folic Acid.*

**Table S4. Predictive factors influencing RBC folate level with multiple imputation (n=519)^1^**

|  | Beta(95%CI) | t | P>\|t\| |
| --- | --- | --- | --- |
| FA supplementation |  |  |  |
| 1.Yes (vs. 0.No) | 0.268(0.011,0.524) | 2.05 | 0.040 |
|  |  |  |  |
| Serum folate(_log_ug/ml) | 0.691(0.527,0.854) | 8.29 | <0.001 |
|  |  |  |  |
| MTHFR C677T |  |  |  |
| 1.CT (vs. 0.CC) | 0.040(-0.138,0.218) | 0.44 | 0.660 |
| 2.TT (vs. 0.CC) | 0.773(0.537,1.008) | 6.45 | <0.001 |
| Cons. | -1.423(-1.757,-1.089) | -8.37 | <0.001 |

Dependent variable: Red blood cell folate level (log-transformed and standardized)

^1^ multiple imputation using chained equations (arbitrary missing pattern with 10 imputations) on missing data of RBC folate(n=2),serum folate(n=6), MTHFR C677T(n=18) was performed (random-number seed: 20230707). The imputations were based on multivariable linear regression(RBC and serum folate) or ordered logistic regression(MTHFR C677T), dependent on remaining covariates (age, sex, BMI, education, occupation, alcohol drinking, smoking, food folate intake, energy intake, folic acid supplementation).

*Abbreviation: FA, Folic Acid; MTHFR, methylenetetrahydrofolate reductase*

**Table S5. Explained variation of RBC folate level in males and females separately**

|  | Beta(95%CI) | t | P>\|t\| |
| --- | --- | --- | --- |
| Males (n=257): |  |  |  |
| FA supplementation |  |  |  |
| 1.Yes (vs. 0.No) | 0.473(-0.076,1.023) | 1.70 | 0.091 |
| Serum folate(_log_ug/ml) | 0.685(0.454,0.915) | 5.86 | <0.001 |
| MTHFR C677T |  |  |  |
| 1.CT (vs. 0.CC) | 0.071(-0.170,0.312) | 0.58 | 0.561 |
| 2.TT (vs. 0.CC) | 0.867(0.053,1.200) | 5.12 | <0.001 |
| Cons. | -1.396(-1.729,-1.06) | -8.23 | <0.001 |
| R-squared | 0.207 |  |  |
| Adjusted R-squared | 0.193 |  |  |
|  |  |  |  |
| Females (n=260): |  |  |  |
| FA supplementation |  |  |  |
| 1.Yes (vs. 0.No) | 0.229(-0.090,0.548) | 1.41 | 0.159 |
| Serum folate(_log_ug/ml) | 0.643(0.385,0.901) | 4.91 | <0.001 |
| MTHFR C677T |  |  |  |
| 1.CT (vs. 0.CC) | -0.040(-0.311,0.231) | -0.29 | 0.771 |
| 2.TT (vs. 0.CC) | 0.729(0.393,1.064) | 4.28 | <0.001 |
| Cons. | -1.287(-1.841,-0.733) | -4.58 | <0.001 |
| R-squared | 0.184 |  |  |
| Adjusted R-squared | 0.171 |  |  |

Dependent variable: Red blood cell folate level (log-transformed and standardized)

*Abbreviation: FA, Folic Acid.*

**Table S6. Dietary nutrient intakes of the study population^1^**

| Dietary nutrient intake | Total (n=519) | Male(n=259) | Female(n=260) | P |
| --- | --- | --- | --- | --- |
| Total energy intake(kcal/d) | 2574(447) | 2805(358) | 2345(407) | <0.001 |
| Carbonhydrate (g/d) | 268(61) | 287(60) | 248(55) | <0.001 |
| Fat (g/d) | 98(27) | 107(26) | 89(25) | <0.001 |
| Protein (g/d) | 155(42) | 172(39) | 138(38) | <0.001 |
| Fiber(g/d) | 24.0(10.6) | 24.5(10.4) | 23.5(10.9) | 0.313 |
| Vitamin A (mg/d) | 2058(1267) | 2130(1291) | 1986(1240) | 0.195 |
| Vitamin B1 (μg/d) | 1.57(0.48) | 1.69(0.47) | 1.45(0.47) | <0.001 |
| Vitamin B2 (mg/d) | 2.32(0.63) | 2.48(0.61) | 2.16(0.61) | <0.001 |
| Folate (mg/d) | 296.9(66.7) | 327.3(63.2) | 266.7(55.4) | <0.001 |
| Niacin (mg/d) | 38.5(10.5) | 42.3(9.6) | 34.6(9.9) | <0.001 |
| Vitamin E (mg/d) | 25.1(9.8) | 26.6(10.1) | 23.7(9.3) | <0.001 |
| Na (mg/d) | 1431(888) | 1597(880) | 1266(867) | <0.001 |
| Ca (mg/d) | 1112(365) | 1174(361) | 1051(358) | <0.001 |
| Fe (mg/d) | 44.6(24.1) | 47.2(23.6) | 42.1(24.3) | 0.016 |
| Vitamin C (mg/d) | 272(115) | 276(113) | 268(116.7) | 0.404 |
| Total Cholesterol (mg/d) | 1076(466) | 1228(450) | 926(433.7) | <0.001 |
| Glycemic Index | 55.1(8.6) | 56.2(8.5) | 54.1(8.6) | 0.005 |
| Glycemic Load | 148.6(43.9) | 162.5(44.3) | 135(39.0) | <0.001 |

^1^ presented as mean(standard deviation)
